# Orientation Measurement Based on Magnetic Inductance by the Extended Distributed Multi-Pole Model

**DOI:** 10.3390/s140711504

**Published:** 2014-06-27

**Authors:** Fang Wu, Seung Ki Moon, Hungsun Son

**Affiliations:** 1 School of Mechanical and Aerospace Engineering, Nanyang Technological University, 50 Nanyang Avenue, Singapore 639798, Singapore; E-Mails: WU0013NG@e.ntu.edu.sg (F.W.); skmoon@ntu.edu.sg (S.K.M.); 2 School of Mechanical and Nuclear Engineering, Ulsan National Institute of Science and Technology (UNIST), UNIST-gil 50, Eonyang-eup, Ulju-gun, Ulsan 689-798, Korea

**Keywords:** mutual inductance, magnetic field, coil, orientation measurement, distributed multiple dipole

## Abstract

This paper presents a novel method to calculate magnetic inductance with a fast-computing magnetic field model referred to as the extended distributed multi-pole (eDMP) model. The concept of mutual inductance has been widely applied for position/orientation tracking systems and applications, yet it is still challenging due to the high demands in robust modeling and efficient computation in real-time applications. Recently, numerical methods have been utilized in design and analysis of magnetic fields, but this often requires heavy computation and its accuracy relies on geometric modeling and meshing that limit its usage. On the other hand, an analytical method provides simple and fast-computing solutions but is also flawed due to its difficulties in handling realistic and complex geometries such as complicated designs and boundary conditions, *etc.* In this paper, the extended distributed multi-pole model (eDMP) is developed to characterize a time-varying magnetic field based on an existing DMP model analyzing static magnetic fields. The method has been further exploited to compute the mutual inductance between coils at arbitrary locations and orientations. Simulation and experimental results of various configurations of the coils are presented. Comparison with the previously published data shows not only good performance in accuracy, but also effectiveness in computation.

## 1. Introduction

Motion sensors, as an essential component in many applications such as automation [1], robotics [2,3], manufacturing machine and medical devices, *etc.*, have been developed to monitor or control electrical and mechanical systems. As growing techniques in integrated electronics and computer during the past decades, many different kinds of sensors with various working principles including pedometers, accelerometers, optical and magnetic sensor, *etc.*, have been commercially available but continue to be researched to improve their performance [4]. Among these sensors, optical/imaging sensors for detecting motion/position have also been widely used since they offer visual identification [5], but their sampling rate is low and thus it is often limited in a fast dynamic system. An ultrasound and radar device can be utilized to monitor wide areas with high accuracy, but it is bulky and cannot be used with obstacles along the optical path [6]. Magnetic induction sensors have been utilized in numerous applications such as position, orientation measurement and path tracking [7–10]. They are compact in size, easy to operate and capable of providing high sensitivity without mechanical contact, making them therefore suitable for small and precise measurement systems, for instance, assisting intubation procedures for medical usage and narrow pipe inspection where the space is limited and orientation should be measured and controlled.

Magnetic inductance has been studied and calculated by a number of researchers. Based on the Neumann formula, Grover's work [11] provides a method to calculate the mutual inductance in the form of a single integral and dictionary matching method has therefore been widely used. With the help of highly developed numerical techniques and advanced computation power, it is now possible to accurately calculate the mutual inductance [12]. However, there is still interest [13–17] to develop analytical or semi-analytical methods in order to simplify the mathematical procedures and reduce the computation time. Most of these works developed the equations based on the Neumann formula, which describes the mutual inductance using only geometric parameters, and addressed the problem utilizing specific geometric relations, such as coaxial [13] and inclined axes in the same plane [16]. Series sum of solutions were usually obtained and able to provide reasonable simulation results along with simplified assumptions.

Recently, advances in computer technologies have led to the development of fast computation along with efficient numerical methods. In particular, magnetic inductance of various shaped coils at their geometric relation has been studied [18,19]. The computation process would require series of integral operations for various shapes and locations of coils. Although the technique has been developed, it is still difficult to apply for many different shapes of coils in various configurations. Any change in the existing form/solution has to be evaluated again accordingly. In addition, computation time and effort are still demanding though analytical formulas and results are available in [20,21]. This could cause an ambiguity problem since various approximations have to be assumed to utilize the analytical solution. To improve the performance, it would be better to have an accurate and fast-computing method to characterize the magnetic field. Unlike existing methods, the distributed multi-pole (DMP) model in [22], based on the analytical expression of electromagnetic field from source/sink, provides compact solutions and offers fast-computation without sacrificing accuracy. However, the existing DMP model is limited to the analysis of static magnetic fields and it is difficult to characterize the time-varying magnetic field directly. To explore the method for time-varying fields, the DMP method is extended by the magnetic vector potential based on the magnetic doublet model referred to as extended DMP (eDMP) model. It offers an effective computation of time-varying magnetic fields and can be extended to magnetic inductance and energy. In the paper, the eDMP model and mutual inductance computation procedure are explained first. Then both simulation and experimental results of various coils and their configuration are compared to demonstrate the modeling accuracy and computational efficiency of the method.

## 2. Magnetic Field Model

### 2.1. Extended Distributed Multi-Pole Model

The DMP model in [22] has been developed to characterize the static magnetic field of a permanent magnet (PM) or an electromagnet (EM). Unlike a single dipole model in [23,24], widely used to model the far field of magnets due to its limited accuracy, the DMP model characterizes the magnetic field near magnets with a set of magnetic source and sink poles. The method offers relatively simple solutions without sacrificing accuracy and thus it can be used for design and control applications requiring fast computation. However the existing DMP model has a limit when analyzing static magnetic fields and is difficult to apply for many practical applications including magnetic inductance, eddy current, *etc.*, since it cannot account for time-varying magnetic fields. To overcome this drawback, the DMP model is further developed into the extended DMP (eDMP) model for time-varying magnetic fields.

Time-varying magnetic fields can be characterized by Poisson's equation with magnetic vector potential. Integrated with the boundary condition that the vector potential vanishes at infinity and current source being localized as well as finite, the closed-form solution can be expressed in Equation (1):

(1) A(r)=μ4π∫J(r')|r−r'|dV'

Where *V′* indicates volume enclosing the current density **J** source at position **r′**; *μ* is permeability. Unlike the DMP model with a set of scalar poles (source/sink), eDMP uses magnetic dipoles, expressed as magnetic dipole moment **m**, which can further simplify Equation (1) to Equation (2):

(2) A(r)=μm×(r−r')4π|r−r'|3

where **r′** indicates location of magnetic dipole.

**Figure 1.**
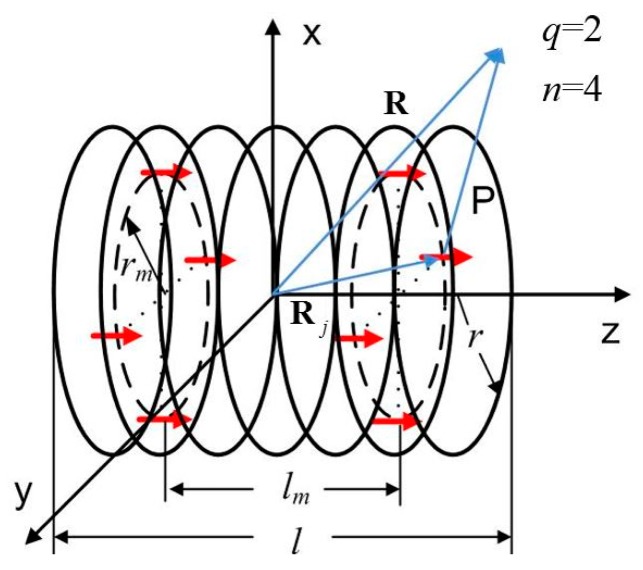
Extended DMP model.

Like the DMP method, the eDMP method as shown in Figure 1 utilizes a set of distributed magnetic dipoles placed in multiple layers to retain the physical shape of a coil. The simulated magnetic field can be then calculated by summing the contributions of each magnetic dipole.

For simplicity, a cylindrical coil is used in Figure 1 as demonstration since it is commonly used in many practical applications, but the method can be applied for any coil shape. Coil geometry is depicted using black circles. Radius (*r*) and length (*l*) of the coil will be geometric references for the eDMP model, indicating where the dipoles should be located. Magnetic dipoles are represented by red arrows and arranged as two circular pattern layers (black dashed lines). The computation process of the eDMP model is analogous to the DMP model in [9]. The magnetic field model can be obtained by minimizing a discrepancy between the modeling results and the reference data, which could be either a computed or measured field. Model parameters of the eDMP model are listed as the follows:

- Number of layers (*q*);
- Radius of each layer (*rm*);
- Distance between positive and negative layers (*lm*);
- Number of dipoles in each layer (*n*);
- Dipole moment (**m**).

As shown in Figure 1, the center of the coil is located at the origin of a coordinate system xyz and magnetization of the coil is parallel to the *z* axis. A number of dipoles are located inside the coil to account for the field source. Vector potential **A** and magnetic flux density **B** outside the coil can be then represented by the contributions of magnetic dipoles:

(3) $$\text{A}\left(\text{r}\right)=\sum_{j=1}^{q\times n}\frac{\mu_{0}{\text{m}}_{j}\times {\text{P}}_{j}}{4\pi {\left|{\text{P}}_{j}\right|}^{3}}$$

(4) $$\text{B}\left(\text{r}\right)=\sum_{j=1}^{q\times n}\frac{\mu_{0}\left[3\left({\text{m}}_{j}•{\text{e}}_{j}\right){\text{e}}_{j}-{\text{m}}_{j}\right]}{4\pi {\left|{\text{P}}_{j}\right|}^{3}}$$

where **P***j*=**r**–**R***j*, **e***j*=**P***j*/|**P***j*|; **r** indicates the location that magnetic field is calculated; **R***j* indicates the location of *j*th dipole, which can be expressed as ${\text{R}}_{j}=(r_{m}\cos\frac{2\pi j}{n},r_{m}\sin\frac{2\pi j}{n},\pm \frac{l_{m}}{2})$ using modeling parameters; **m***j* is the moment of *j*th magnetic dipole; *q*×*n* is the number of the dipoles. The dipoles are placed along the circumference since the physical shape of the coil is a cylinder. In addition, polarization of the coil is along the *z* axis, utilizing a magnetic dipole with its direction parallel to the *z* axis, indicating *mx*=0 and *my*=0. Magnetic field in Equation (1) along the *z* axis can be calculated and used as the reference field when assuming current flows only within the *xy* plane Figure 1. Error *E* can be expressed in Equation (5):

(5) E(r)=∫02l(|B(r)−B'(r)|2)dz

where **B**(**r**) is the simulation result obtained from Equation (4) and analytical solution **B′**(**r**) is calculated by **B′**(**r**)= ∇×**A**(**r**) from Equation (2). The integral was done along the *z* axis from the central surface to a distance of twice the coil length. Equation (5) is only dependent on the radial location and is usually examined at the outer surface of coil since the eDMP model magnetic field outside the coil.

In order to improve the modeling accuracy and the computation efficiency, additional constraints of magnetic vector potential at finite points can be computed as in Equation (6):

(6) $$\left[\begin{array}{c} \chi_{1}^{T}\\ \delta_{1}^{T}\\ \vdots \\ \chi_{n}^{T}\\ \delta_{n}^{T} \end{array}\right]\left[\text{m}\right]=\left[\begin{array}{c} A_{x1}\\ A_{y1}\\ \vdots \\ A_{xn}\\ A_{yn} \end{array}\right]$$

where $\chi_{ij}=\frac{\mu_{0}\left(y_{mj}-y_{fi}\right)}{4\pi R}$; $\delta_{ij}=\frac{\mu_{0}\left(x_{fi}-x_{mj}\right)}{4\pi R}$; $\chi_{i}^{T}=\left[\begin{array}{ccc} \chi_{i1} & \cdots & \chi_{in} \end{array}\right]$; $\delta_{i}^{T}=\left[\begin{array}{ccc} \delta_{i1} & \cdots & \delta_{in} \end{array}\right]$; $[\text{m}]={\left[\begin{array}{ccc} m_{z1} & \cdots & m_{zn} \end{array}\right]}^{T}$; *Axi* and *Ayi*. (*I* = *1,2,..,n*) represent analytically calculated *x* and *y* components of the vector potential, respectively.

Modeling accuracy in the eDMP model would be affected by the coil geometry, accounted mainly by parameters *rm* and *lm* as well as the number of dipoles, *n* and *q*. The general procedures of the eDMP can be summarized as shown below:

Step 1. Compute and analytically along the magnetization vector from Equations (1) and (2), respectively.
Step 2. Generate an initial set of spatial grid points (*q* and *n*).
Step 3. Formulate (3) and (4) in terms of the unknowns *lm* and **m**.
Step 4. Find *lm* and **m** by minimizing Equation (5) subject to the constraint Equation (6), where **B**(**r**) is obtained from Equation (4). Error computed by Equation (5) is saved.
Step 5. Check the error (5). If Equation (5) is not satisfied, increase *q* or *n*, and repeat from Step 3. Once Equation (5) is satisfied, the optimal parameters *q*,*n*,*lm* and **m** obtained by minimizing Equation (5) using Step 4.

### 2.2. Mutual Inductance

**Figure 2.**
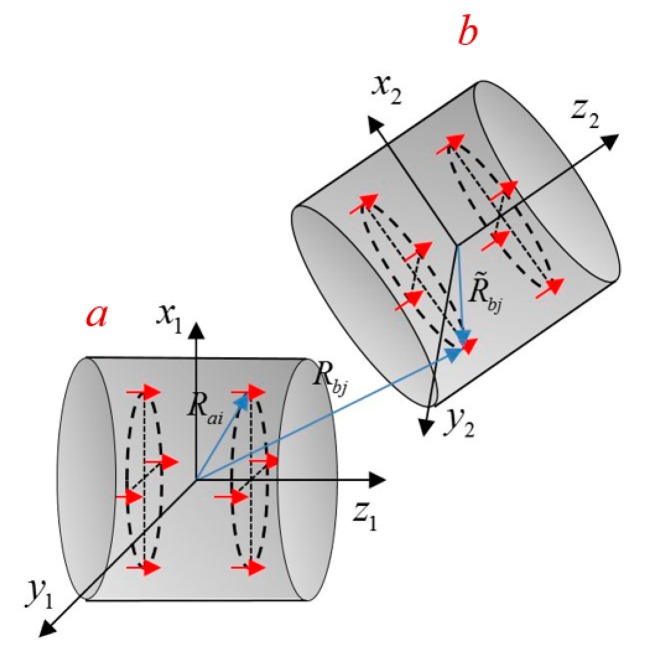
Two coils in their own coordinate systems.

The eDMP model provides a compact solution of the magnetic field of a cylindrical coil. The method can be used to compute the mutual inductance between multiple coils since it effectively accounts for the coil geometry. Figure 2 shows two eDMP models, corresponding to two arbitrarily located coils. Two coordinate systems are fixed with coil *a* and coil *b* respectively. Geometrical relations between dipoles are depicted using blue arrows in Figure 2.

The mutual inductance between coil *a* (exciting current input) and coil *b* (inducing voltage) can be expressed in Equation (7):

(7) $$M_{ab}=\frac{\Phi_{ab}}{I_{a}}=\frac{\int \left(\text{B}•{\text{n}}_{b}\right)dS_{b}}{I_{a}}$$

Where Φ*ab* indicates the magnetic flux in the coil *b* excited by current input *Ia*. By multiplying both the numerator and denominator with a term *Ib* and integrating the magnetic moment of a current loop, the eDMP model replaces the integral operator in Equation (7) with sum of magnetic dipole as below:

(8) $$M_{ab}=\frac{\int \left(\text{B}•I_{b}dS_{b}{\text{n}}_{b}\right)}{I_{a}I_{b}}=\frac{A_{b}\sum_{j=1}^{k}{\text{m}}_{bj}•{\text{B}}_{aj}}{I_{a}I_{b}}$$

where *Ab* indicates the cross-section area of the coil *b*; *k* is the quantity of dipoles for the coil *b*; **m***bj* is the magnetic moment of the *j*th dipole representing coil *b*; **B***aj* is the magnetic flux density at the location of *j*th dipole, calculated in Equation (9):

(9) $${\text{B}}_{aj}=\frac{\mu_{0}}{4\pi }\sum_{i=1}^{p}\frac{3\left({\text{m}}_{ai}•{\text{e}}_{aj}\right){\text{e}}_{aj}-{\text{m}}_{ai}}{{\left|{\text{P}}_{aj}\right|}^{3}}$$

Where **e***aj*=**P***aj*/|**P***aj*|; **P***aj* = **P***aj*=**R***bj*–**R***ai*; **R***bj* indicates the location of *j*th dipole in the *xyz1* coordinate system, **R***bj*=**T**× **R̃***bj*+P0; **R̃***bj* is the dipole position in the *xyz2* coordinate; **P**0 is the origin location of the *xyz2* coordinate in the *xyz1* coordinate; **T** is the coordinate transformation matrix from frame *xyz2* to *xyz1*; *p* is the number of dipoles for the coil *a*.

By substituting Equation (9) into Equation (8), mutual inductance between coil *a* and coil *b* can be computed in Equation (10):

(10) $$M_{ab}=\frac{\mu_{0}A_{b}}{4\pi }\sum_{j=1}^{k}\frac{{\text{m}}_{bj}}{I_{b}}•\sum_{i=1}^{p}\frac{3\left(\frac{{\text{m}}_{ai}}{I_{a}}•{\text{e}}_{aj}\right){\text{e}}_{aj}-\frac{{\text{m}}_{ai}}{I_{a}}}{{\left|{\text{P}}_{aj}\right|}^{3}}$$

where $\frac{{\text{m}}_{bj}}{I_{b}}$ and $\frac{{\text{m}}_{ai}}{I_{a}}$ are dipole moments per current input indicating geometry of two coils since **m** is directly proportional to the current input. Thus, the mutual inductance is only affected by the relative location of coils.

### 2.3. Bended Tubes

**Figure 3.**
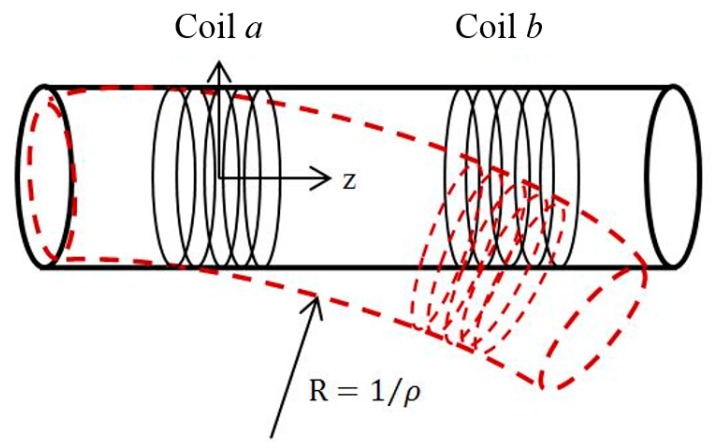
Bending of a flexible tube.

The mutual inductance between two arbitrarily located coils can be calculated using Equation (10). An illustrative example to demonstrate usefulness of the eDMP is shown using a flexible tube for orientation measurements. Figure 3 shows the flexible tube with two coils.

Black solid lines indicate the tube without any bending and red dot lines after bending. The bending curvature of represented as *ρ* and the radius of the curve is expressed as R. An input current is applied at the coil *a* and orientation of the coil *b* can be estimated by measuring an induced voltage in coil *b*.

The reference coordinate is set at the central of the coil *a* as shown in Figure 3. The orientation of the coil *b* with respect to the coil *a* can be presented by the radius of the inner arc *R* and the corresponding curvature *ρ*. It is convenient to express the curvature for changing the tube orientation since the arc distance between two coils remains the same. In addition, the coil winding might be bent along with the tube since the tube is soft so that each layer of winding has a different orientation. It has a major influence to the magnetic field and mutual inductance of the coil *b* although the coil deformation could be small. In order to account the smooth curvature of bending, the dipole moments are continuously computed according to the bending curvature. **m***ai* and **m***bj* in Equation (10) can be expressed as below:

(11a) $${\text{m}}_{ai}={\text{T}}_{a}{\text{m}}_{ao,i}$$

(11b) $${\text{m}}_{bj}={\text{T}}_{b}{\text{m}}_{bo,j}$$

where ${\text{T}}_{a}=\left[\begin{array}{ccc} \cos\left(l_{ma}\rho \right) & 0 & \sin\left(l_{ma}\rho \right)\\ 0 & 1 & 0\\ -\sin\left(l_{ma}\rho \right) & 0 & \cos\left(l_{ma}\rho \right) \end{array}\right]$, ${\text{T}}_{b}=\left[\begin{array}{ccc} \cos\left(l\rho \mp l_{mb}\rho \right) & 0 & \sin\left(l\rho \mp l_{mb}\rho \right)\\ 0 & 1 & 0\\ -\sin\left(l\rho \mp l_{mb}\rho \right) & 0 & \cos\left(l\rho \mp l_{mb}\rho \right) \end{array}\right]$. **T***a* and **T***b* represent the transformation matrix for the coil *a* and *b* respectively; **m***ao,i* and **m***bo,j* are the original dipole moments; *l* indicates the arc length between the two coils, *ρ* represents the curvature, *lm* indicates the distance between positive and negative layers.

For simplicity, Equation (9) can be expressed in the matrix form as below:

(12) $${\text{B}}_{aj}=\frac{\mu_{0}}{4\pi }\sum_{i=1}^{p}\text{F}{\text{m}}_{ai}$$

where $\text{F}=\left[\begin{array}{ccc} 3Px_{j}^{2}-1 & 3Py_{j}Px_{j} & 3Pz_{j}Px_{j}\\ 3Px_{j}Py_{j} & 3Py_{j}^{2}-1 & 3Pz_{j}Py_{j}\\ 3Px_{j}Pz_{j} & 3Py_{j}Pz_{j} & 3Pz_{j}^{2}-1 \end{array}\right]$, describing the relation between magnetic field and dipole moment in matrix form; *Px*, *Py*, *Pz* indicates the x, y, and z components of **P***aj*=**T**× **R̃***bj*+P0–**R̃***ai*; **B***aj* represents the magnetic field at the location of the *j*th dipole of the coil *b*.

The mutual inductance between two coils can be computed by substituting Equations (11) and (12) into Equation (8), expressed as the matrix multiplication form:

(13) $$M_{ab}=\frac{A_{b}}{I_{a}I_{b}}\sum_{j=1}^{k}\sum_{i=1}^{p}{\left({\text{T}}_{b}{\text{m}}_{bo,j}\right)}^{\text{T}}\text{F}{\text{T}}_{a}{\text{m}}_{ao,i}$$

Where **m***ao,i* and **m***bo,j* indicate the *i*th and *j*th dipole moment of the coil *a* and *b* respectively; **T***a* and **T***b*. represent the transformation matrix in (11); **F** given in Equation (12) including curvature. Since the axes of two coils are initially along the *z* axis, there is only one nonzero term in the dipole moment matrix **m***ao,i* and **m***bo,j*. The mutual inductance can be simply computed by considering z-components.

## 3. Simulation and Experiments

The eDMP model is demonstrated with three examples; magnetic flux density of the coil, mutual inductance between two coils, and orientation curvature of a flexible tube. Simulation and experimental results are provided to evaluate the performance.

### 3.1. Magnetic Flux Density

**Figure 4.**
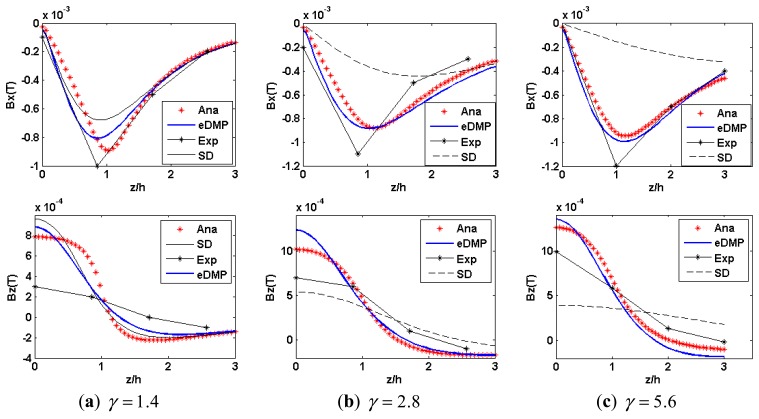
Simulation results of different aspect ratio.

Time-varying magnetic fields of two different shaped coils with different aspect ratio (γ=2*r*/*l*) are simulated by three different methods; eDMP, Single Dipole model, and an analytical solution. The Single Dipole model is well known and commonly used for many applications due to its simplicity. In order to examine how the coil geometry affects the modeling accuracy, the coil length is fixed at 7 mm, which corresponds to 20 turns of winding. Coils with different radii, 0.5 cm, 1 cm, and 2 cm, are compared. The model parameters of the eDMP are detailed in Table 1 and the simulated magnetic field is shown in Figure 4.

For coil *a*, the maximum modeling error using Single Dipole (SD) model is 12% and that of eDMP is 5.8%, which indicates improved modeling accuracy. As the aspect ratio increases to 2.8 and 5.6 in Figure 4b,c, respectively, the modeling error using SD model is getting larger and cannot simulate the magnetic field of flat coils. On the contrary, the eDMP model provides a modeling discrepancy of 9.34% and 13.41% for the coil *b* and *c*, respectively due to its capability of accounting for the coil shape. The actual magnetic field is also measured and shown in Figure 4 to validate the analytical model and eDMP model, similar to each other. The measured magnetic field can also be used as the reference data for eDMP model in Equation (5) to improve accuracy if necessary.

### 3.2. Mutual Inductance Simulation

Two examples in [21] are used to simulate mutual inductance between two coils, including different combinations of thin wall solenoids and filamentary circular coils.

#### 3.2.1. Thin Wall Solenoid and Filamentary Circular Coil

**Figure 5.**
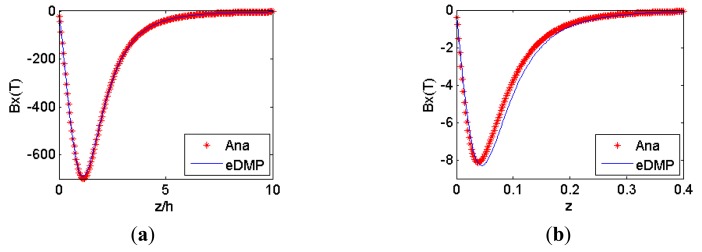
Simulation results: (**a**) coil *a*; (**b**) coil *b*.

Two coils are modeled using the eDMP method, and the eDMP parameters are detailed in Table 2. Magnetic field simulation results of the eDMP method are compared to the analytical model in Figure 5 and the maximum error of coil *a* is 3.66% and coil *b* is 6.8%.

**Figure 6.**
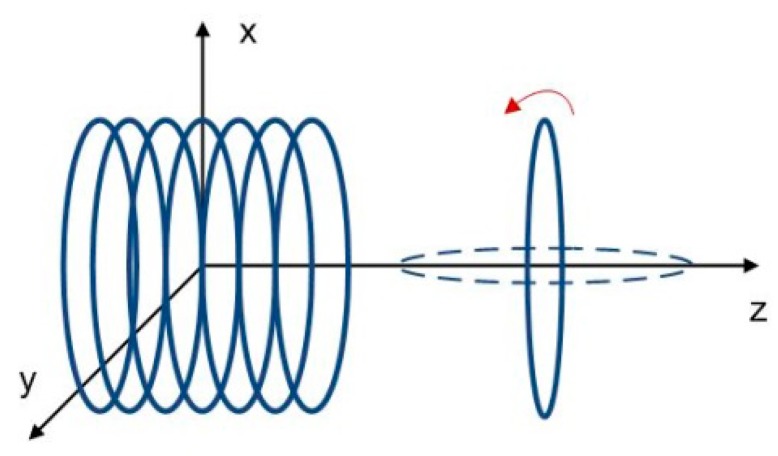
Multi and single wound coils.

**Figure 7.**
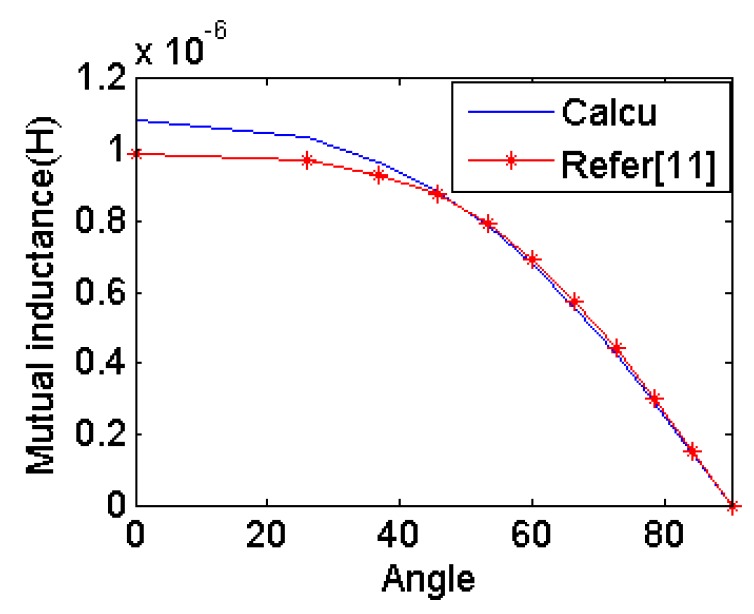
Mutual inductance simulation results.

Two coils are located coaxially and distance between the two centers is 12 cm as shown in Figure 6. The orientation of the circular coil is rotated along the *y* axis. Mutual inductance is calculated by the eDMP model and the method in [11]. Figure 7 shows the results for each orientation and the mutual inductance agrees with each other and the maximum error is 9.8%.

#### 3.2.2. Two Thin Wall Solenoids

**Figure 8.**
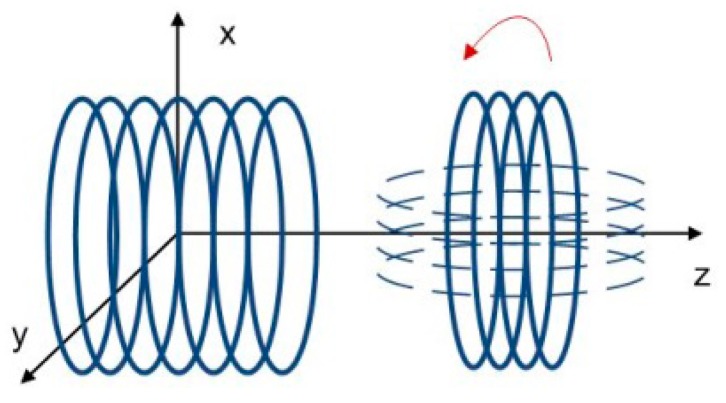
Multi and multi wound coils.

**Figure 9.**
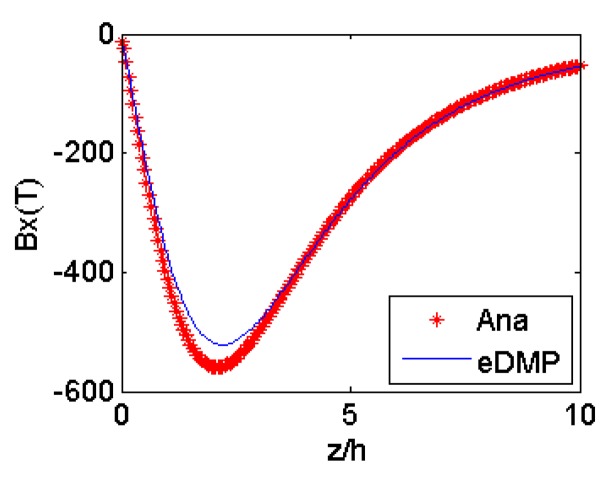
Field simulation results of coil *b*.

Mutual inductance of two multi-wound coils shown in Figure 8 similar to Figure 6. The detailed parameters are given in Table 3. Simulation results are also shown in Figure 9 and the maximum error is 10.2%.

**Figure 10.**
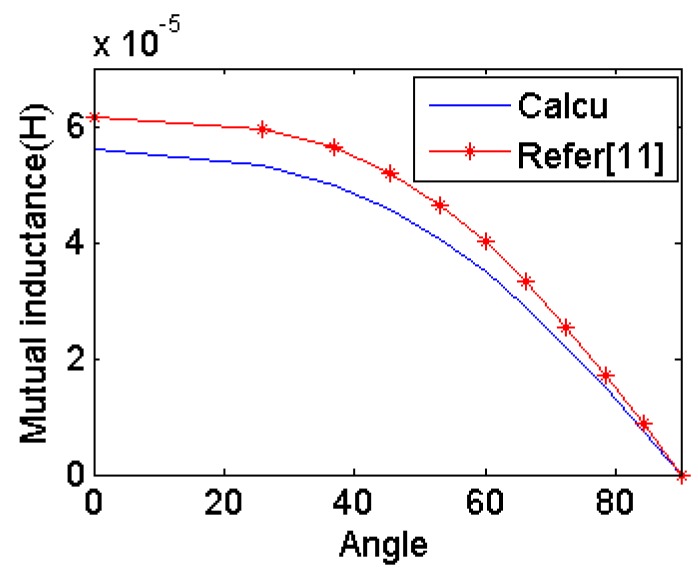
Mutual inductance simulation results.

Figure 10 shows comparison of the mutual inductance compared with published data in [11]. The maximum error is 8.9%.

#### 3.2.3. Orientation of Coils along a Flexible Tube

**Figure 11.**
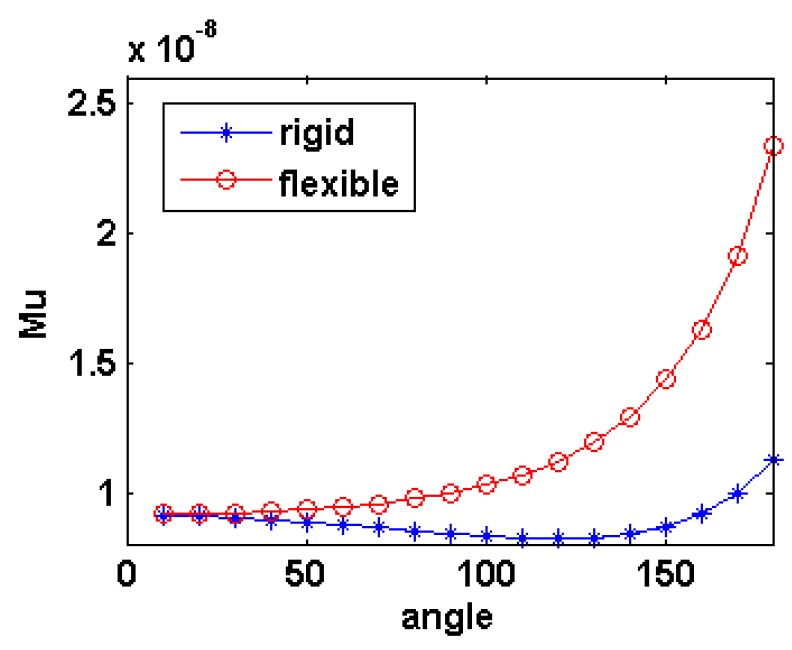
Comparison of simulation results.

Mutual inductance of coils between rigid and flexible tube are simulated and compared in Figure 11. Unlike the rigid tube, the flexible tube includes different orientations along each coil wound. Variance of two mutual inductances can be shown when the tube is bended 20° from Equations (10) and (15). This indicates the coil deformation has little effect as long as the bending angle is small. However, the discrepancy becomes much more apparent as the angle increases further.

### 3.3. Experimental Results

**Figure 12.**
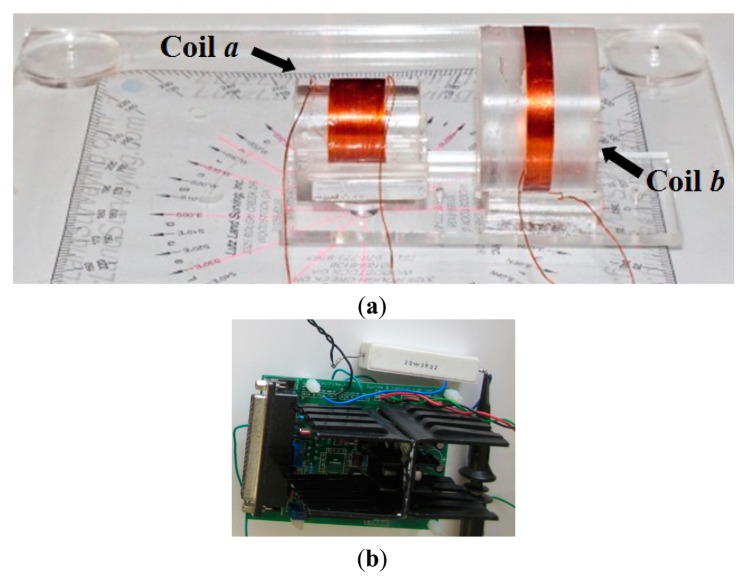
Experimental set-up: (**a**) the base and rotating structure; (**b**) the power amplifier circuit.

Figure 12 shows an experimental setup of two coils capable of rotating their orientation. The coil *a* is fixed at the center, but the other coil *b* can be adjusted in distance and rotated with respect to coil *a*. The base structure and rotating plate are made of non-conductive, transparent acrylic. The winding wires are connected to instruments using a BNC cable with clips. Because the resistance of coil *a* is below 1 Ω, the current input from function generator is amplified through a power amplifier circuit and applied to the excitation coil, which is in a series circuit with a high power resistor. The amplifier circuit has a bandwidth of 1 MHz, and provide an adjustable gain. An oscilloscope with a NI-USB6366 DAQ device is used to measure to analyze the signal due to small magnitude of measurement.

**Figure 13.**
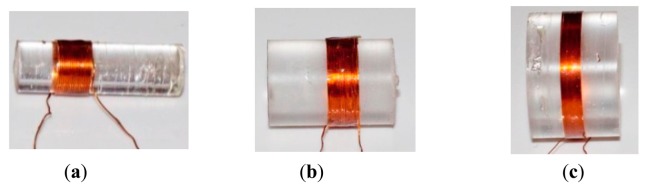
Coil designs: (**a**) coil 1 (*γ*=1.4); (**b**) coil 2 (*γ*=2.8); (**c**) coil 3 (*γ*=5.7).

In addition, three different coils with different aspect ratio (*γ*) are tested as in Figure 13. Input current 0.5 A of 10 kHz is applied through the coil *a*, design of which is as coil 1 in Figure 13, and different configurations are used for coil *b*. The experimental parameters are summarized in Table 4. In addition, the conventional Single Dipole (SD) model is also computed for comparison purpose.

#### 3.3.1. Coil 1:1 cm Diameter, 20 Turns

**Figure 14.**
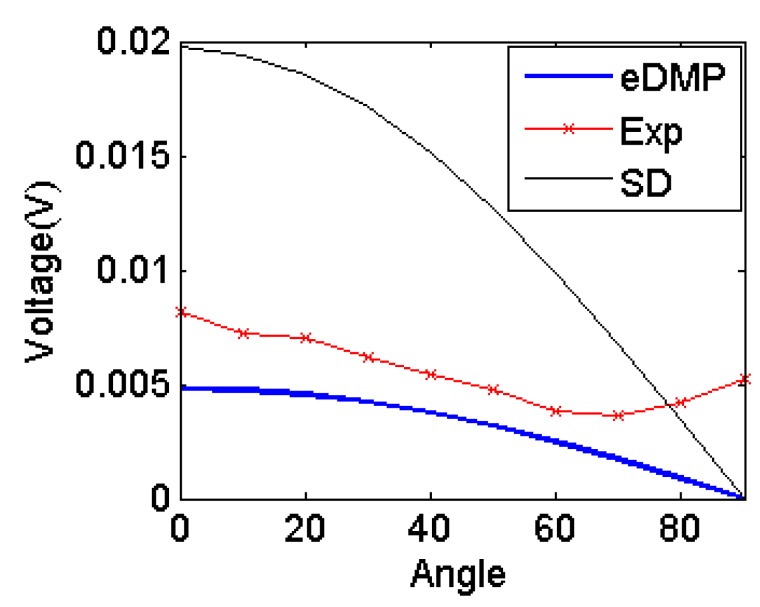
Simulation results comparison.

Figure 14 shows the induced output voltage due to excitation the coil *a* becomes as small as 5 mV when the orientation is bigger than 60°, almost the same magnitude as noise. As the orientation angle further increases, the signal would further decrease and noise would become the dominant, indicating the designed coil can only be operated in a smaller range than 60°.

#### 3.3.2. Coil 2: 2 cm Diameter, 20 Turns

**Figure 15.**
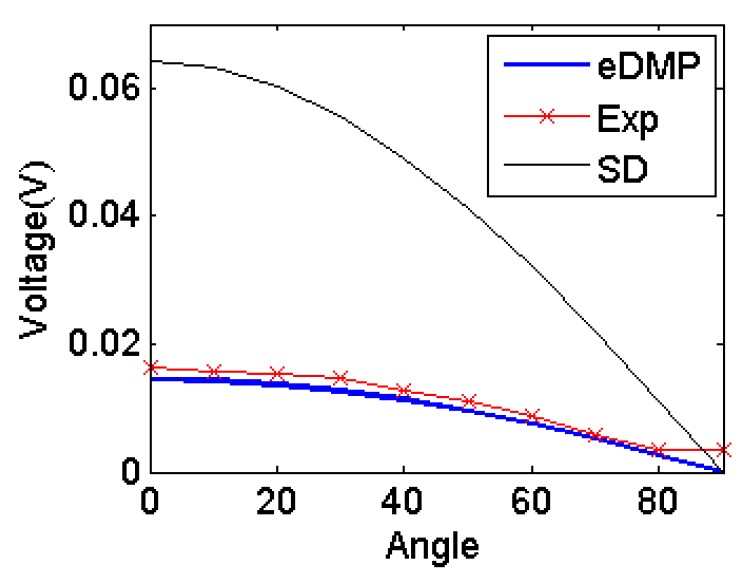
Simulation results comparison.

The result in Figure 15 shows that the eDMP model provides more accurate results than the SD model. The maximum error between the simulations and experiment is only 0.0017 V, around 9% of the experimental measurements. A signal amplifier gain is 100 to measure higher output from the sensing coil and improve the signal and noise ratio.

#### 3.3.3. Coil 3: 4 cm Diameter, 20 Turns

**Figure 16.**
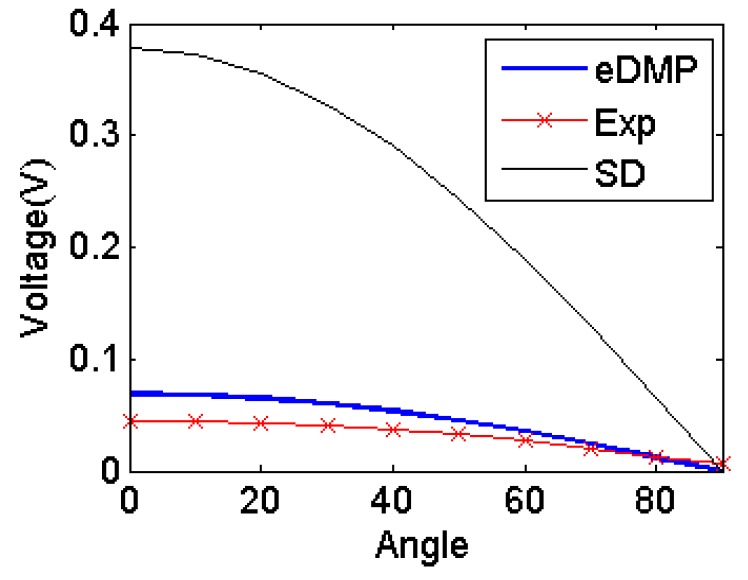
Simulation results comparison.

Similarly, Figure 16 shows the errors are getting bigger as the coils become a flat and multiple wound. However, the error is still much smaller as compared to the SD model. The maximum difference between the experimental results and eDMP model is 0.02 V. The error is expected due to the eDMP error for coils with larger aspect ratio (*γ*=2*a*/*h*).

### 3.4. Tube Experiment

**Figure 17.**
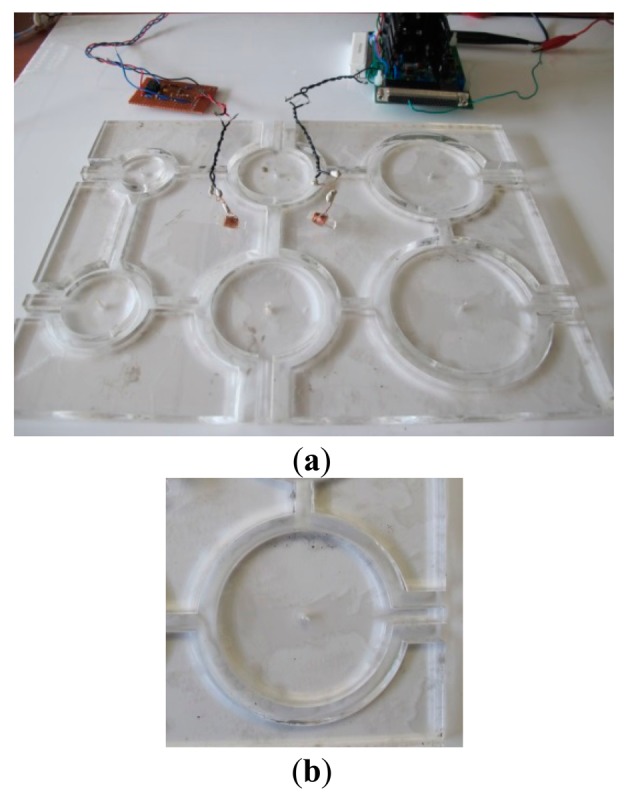
Experimental setup: (**a**) Tube experiment set-up; (**b**) Single arc.

**Figure 18.**
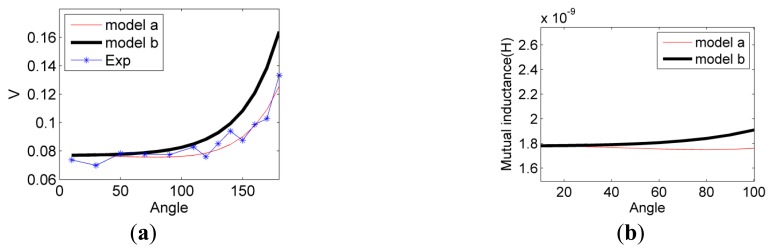
Results of arc distance to be 2.5 cm: (**a**) Comparison of experiment and simulation; (**b**) A closer look of two models.

Two coils of 20 turns are wound along with small solid cylinders (no bending) with 5 mm diameter as shown in Figure 17 and the diameter of copper wire is 0.3 mm. Arc distances with 2.5 cm are fabricated as a guide of the coil path. The eDMP model simulates two coils with and without smooth curvature and referred to as models (a) and (b), respectively. Similarly, the current input of the coils is 1 Amp with 10 kHz. The output voltage of the other coil and mutual inductance are compared in Figure 18.

The results show the difference of modeling with and without bending, although the error is relatively small. However, as angle range 10°–100° were examined closely as in Figure 18b, the output voltage of model (a) is decreasing while model (b) is increasing, which made the model (b) share the same trend with experimental results.

## 4. Conclusions

The paper presents a general and fast-computing method to calculate mutual inductance between coils. The time-varying magnetic field of the coils is first characterized by an extended distributed multi-pole (eDMP) model and then the mutual inductance is further computed by discretizing the integral with discrete magnetic dipoles. The eDMP method offers a simple form of summation to estimate the mutual inductance. Simulation and experimental results of various designed coils and configurations showed the improvement in modeling accuracy of the eDMP model.

Besides computing efficiency and accuracy, the eDMP model is capable of simulating arbitrary shaped coils and computing the magnetic field in real-time. For demonstration of the eDMP method, coil orientations in 2D space have been discussed here for simplicity, but the method can be expanded to orientations in 3D space without any difficulty. The simulation and experimental results showed an excellent agreement to each other. In addition, the accuracy has been improved compared to the conventional single dipole as well as the analytical method. The method presented in the paper can be applied to developing orientation sensors in various applications such as path tracking of a medical intubation tube. The human-friendly nature of inductive phenomenon indicates a huge potential in many areas.

## Figures and Tables

**Table 1.** eDMP model parameters.

**Coil Geometry**	**DMP Model**
*r*=0.5 *cm* *l* =0.7 *cm* 20 turns	*q* = 2, *n* = 6 *lm* =0.3 *cm* *rm* =0.25 *cm* |**m**| =2.11×10–4
*r* =1 *cm* *l* =0.7 *cm* 20 turns	*q* = 1, *n* = 6 *lm* =0 *rm* =0.61 *cm* |**m**| =0.0018
*r* =2 *cm* *l* =0.7 *cm* 20 turns	*q* = 1, *n* = 6 *lm* =*0* *rm* =1.46 *cm* |**m**| =0.0048

**Table 2.** eDMP model parameters.

	**Coil Geometry**	**DMP Model**
Coil *a*	a solenoid with 6 cm radius, 12 cm height, and 120 turns of winding	*q* = 1, *n* = 6
		*lm*=5.88 *cm*
		*rm*=3 *cm*
		|**m**| =1.8825

Coil *b*	a circular coil with radius to be 5 cm	*q* = 1, *n* = 6
		*lm*=0
		*rm*=2.5 *cm*
		|**m**| =0.0303

**Table 3.** DMP model parameters.

	**Coil Geometry**	**DMP Model**
Coil *a*	a solenoid with radius to be 6 cm, height to be 12 cm, and the number of turns to be 120	*q* = 1, *n* = 6
		*rm*=3 *cm*
		*lm*=5.88 *cm*
		|**m**| =1.8825

Coil *b*	a solenoid with radius to be 5 cm, height to be 4 cm, and the number of turns to be 60	*q* = 1, *n* = 6
		*lm*=0
		*rm*=2.5 *cm*
		|**m**| =1.5645

**Table 4.** Experiment parameters.

Frequency		10 kHz
Current		0.5 A (RMS)

Coil *a*	Diameter	2 cm
	Turns	40
	Resistance	0.5 Ω

Coil *b*		Coil 1,2 and 3 in Figure 13

## Nomenclature

**Upper Characters**
**A**: Magnetic vector potential
*A*: Cross-section area of a coil
**B**: Magnetic flux density
*I*, **J**: Current, Current density
*M*ab: Mutual inductance between coil a and b
*R*: Radius of the inner arc when the tube is bent
**R***j*: Position vector of the *j*th dipole
**T**: Coordinate transformation matrix

**Greek Characters**
Φ*ab*: Magnetic flux in coil *b* excited by coil *a*
*μ*, *μ*0: Permeability, Permeability of air
*ρ*: Curvature of the bending tube

**Lower Characters**
**r**: Position vector of the magnetic field
**r′**: Position vector of the current density
*r*: Radius of the coil
*l*: Length of the coil
*rm*: Radius of each layer in the eDMP
*lm*: Distance between the positive and negative layers
*n*: Number of dipoles in each layer
*q*: Number of layers in the eDMP model
**m***j*: Magnetic moment of the *j*th dipole
*p*: Number of dipoles for coil a
*k*: Number of dipoles for coil b
